# Recommendations for Developing Support Tools With People Suffering From Chronic Obstructive Pulmonary Disease: Co-Design and Pilot Testing of a Mobile Health Prototype

**DOI:** 10.2196/16289

**Published:** 2020-05-15

**Authors:** Alan Davies, Julia Mueller, Jean Hennings, Ann-Louise Caress, Caroline Jay

**Affiliations:** 1 School of Health Sciences University of Manchester Manchester United Kingdom; 2 Manchester Academic Health Science Centre Manchester United Kingdom; 3 School of Human and Health Sciences University of Huddersfield Huddersfield United Kingdom; 4 Department of Computer Science University of Manchester Manchester United Kingdom

**Keywords:** chronic obstructive pulmonary disease, app design, mHealth, ecological momentary assessment, mobile phone

## Abstract

**Background:**

Gaps exist between developers, commissioners, and end users in terms of the perceived desirability of different features and functionalities of mobile apps.

**Objective:**

The objective of this study was to co-design a prototype mobile app for people with chronic obstructive pulmonary disease (COPD). We present lessons learned and recommendations from working on a large project with various stakeholders to develop a mobile app for patients with COPD.

**Methods:**

We adopted a user-centered, participatory approach to app development. Following a series of focus groups and interviews to capture requirements, we developed a prototype app designed to enable daily symptom recording (experience sampling). The prototype was tested in a usability study applying the *think aloud* protocol with people with COPD. It was then released via the Android app store, and experience sampling data and event data were captured to gather further usability data.

**Results:**

A total of 5 people with COPD participated in the pilot study. Identified themes include familiarity with technology, appropriate levels for feeding back information, and usability issues such as manual dexterity. Moreover, 37 participants used the app over a 4-month period (median age 47 years). The symptoms most correlated to perceived well-being were *tiredness* (*r*=0.61; *P*<.001) and *breathlessness* (*r*=0.59; *P*<.001).

**Conclusions:**

Design implications for COPD apps include the need for clearly labeled features (rather than relying on colors or symbols that require experience using smartphones), providing weather information, and using the same terminology as health care professionals (rather than simply lay terms). Target users, researchers, and developers should be involved at every stage of app development, using an iterative approach to build a prototype app, which should then be tested in controlled settings as well as *in the wild* (ie, when deployed and used in real-world settings) over longer periods.

## Introduction

### Context

An estimated 65 million people worldwide and 1.2 million people in the United Kingdom alone [1] have chronic obstructive pulmonary disease (COPD). COPD is projected to become the third leading cause of death by 2030 [2,3]. COPD entails a significant personal, economic, and societal burden [4].

COPD patients are predominately older adults, (ex)smokers, and from lower socioeconomic backgrounds [5]. Strategies are needed to help patients manage and monitor their condition over time and between medical assessments to ensure effective long-term management [6]. Importantly, COPD tends to occur alongside various comorbidities (such as coronary heart disease, lung cancer, anxiety, depression, and osteoporosis) due to shared risk factors (eg, aging and smoking) and shared underlying pathophysiological mechanisms [7]. This increases the disease burden, worsens patients’ prognosis, and leads to increased health care costs [7].

### Background

The Department of Health in the United Kingdom has issued recommendations for the prescription of apps as part of the care strategy for various long-term conditions, such as COPD [8]. As COPD patients may not recall the small daily fluctuations in their lung symptoms due to the highly symptomatic nature of the disease, telemonitoring via mobile health (mHealth) technologies may facilitate early intervention through daily monitoring of symptoms [2].

mHealth technologies have been shown to reduce costs associated with long-term COPD management [2]. Qualitative insights show that using mHealth to complement regular care is acceptable to both COPD patients and their health care professionals [9]. The advantages of Web-based health interventions include cost-effectiveness, round-the-clock availability, customizability to personal preferences, and anonymity (when compared with face-to-face interactions) [10]. However, mHealth apps also entail issues and risks, such as a lack of quality control and lack of evaluations of their effectiveness, and privacy and security risks [10-12]. A systematic review of mobile apps used for self-management of chronic conditions concluded that apps can potentially improve health outcomes in long-term conditions through improved symptom management [13]. A systematic review and meta-analysis on randomized controlled trials (RCTs) assessing mobile apps for COPD self-management found evidence for a lower risk of hospital admissions among app users as compared with usual care [14]. A further systematic review and meta-analysis of RCTs found significant improvements in health-related quality of life across 557 COPD patients who used smart technology compared with face-to-face or written support [15]. However, the evidence stemmed from only three studies and was deemed to be of poor quality [15]. Moreover, a review of relevant literature on apps as well as apps available in app stores for people with COPD identified a scarcity of published literature regarding the effectiveness of the apps [2]. There is a clear need for quality-controlled, effective, and acceptable health apps if mHealth is to play a significant role in efforts to prevent and manage diseases.

### Objectives

This study aimed to provide in-depth insights into the views of people with COPD and their caregivers regarding the use of apps in COPD, highlighting key topics and issues around usability that need to be taken into consideration during app development.

## Methods

### Study Context

This study formed part of the large, multistakeholder project *CityVerve*. The CityVerve initiative resulted in the city of Manchester receiving Innovate UK funding for a 2-year period to work on a range of initiatives to apply technology to four separate use cases in partnership with the National Health Service, industry, and universities. The use cases comprised health and social care, transport and travel, culture, and energy and environment. One of the focuses of the health and social care case was COPD. The use of mHealth with this demographic was considered, and a mobile app was developed using a co-design approach and prototyping with participants who were diagnosed with COPD. Project members included academic researchers, software engineers, and health care professionals.

### Study Design

The approach taken in this study is similar to the *iterative convergent mixed-methods design* proposed by Alwashmi et al [16]. The authors propose the use of a mixed methods framework in which both qualitative and quantitative data collection and analysis are used in iterations to develop mHealth interventions and enhance the usability of such interventions [16].

### Ethical Approval

Ethical approval for the study was granted by the University of Manchester (2017-2941-4477). An overview of the main phases can be seen in Textbox 1.

**Overview of the project phases.**

Phase 1: Patient and public involvement work and prototyping

- Aim: To co-design a prototype app with chronic obstructive pulmonary disease (COPD) patients and caregivers
- Methods: Co-design, paper prototyping, focus groups, and direct observation
- Deliverables: A first version digital of the digital prototype on a mobile device
- Participants: approximately 48

Phase 2: Usability study

- Aim: To see how people would actually use the app and identify any usability issues
- Methods: Think Aloud, interviews, System Usability Scale (survey), and observations
- Deliverables: Data on user feedback, survey results, and update prototype with results
- Participants: 5

Phase 3: Wider deployment and usability testing

- Aim: To test the app with a wider number of COPD patients not directly involved in its design
- Methods: Experience sampling, event data collection, and analysis
- Deliverables: User event data
- Participants: 37

### Procedure

Development and evaluation of the app took place during the three main phases. The *first phase* consisted of patient and public involvement (PPI) work, leading to the development of *mock-up* app designs and subsequently a prototype app. PPI work was carried out by attending and hosting a number of events for COPD patients and their families. We also invited members of a COPD self-help group to attend several sessions to discuss their needs and preferences. Together with participants, we developed paper prototypes for the app. The main stakeholders involved in the project were patients, families, caregivers, and members of the CityVerve project team. As this phase constituted PPI work rather than research, sessions were not audio recorded. Notes were taken following discussion and observation of the participants.

The *second phase* involved a usability study with the prototype app, using the “think aloud” paradigm. Participants were instructed to set up the app, browse its features, and verbalize their thoughts about the app as they proceeded. Participants then completed the System Usability Scale (SUS) questionnaire [17]. The “think aloud” sessions were audio recorded and transcribed. In addition, 2 researchers attended each session to observe and record notes. Transcripts and observation notes were subsequently analyzed thematically using framework analysis [18]. This is a qualitative method where themes are represented in columns and participants in rows, with participants’ responses summarized in the associated theme’s column (Multimedia Appendix 1). This facilitates comparison across participants for the respective themes. All data were coded by 1 researcher, and a random subselection of quotations was coded by a second independent researcher to test for coding agreement between the reviewers (interrater reliability). Cohen kappa indicated substantial agreement (84.6% agreement; κ=0.75).

The *third phase* involved deploying the app on the Google Play store for a 4-month period to collect the self-reported symptom data and event data to evaluate the app over a longer period with a wider group of the target population. The app was advertised by disseminating a 150-word summary of the study (or in the case of Twitter, a 220-character summary) to relevant groups on social media, various mailing lists, websites, and newsletters, with a link to the app’s playstore page. For mailing lists, newsletters, websites, and Twitter, we approached the relevant admin who sent the information out or posted it to their website and Twitter account. For Facebook groups, we first approached the admin to obtain permission before posting. Targeted groups were either COPD-related (eg, COPD support groups) or likely to include an older demographic (eg, aging-related mailing lists) because COPD mostly affects older adults [19].

### The App

**Figure 1:**
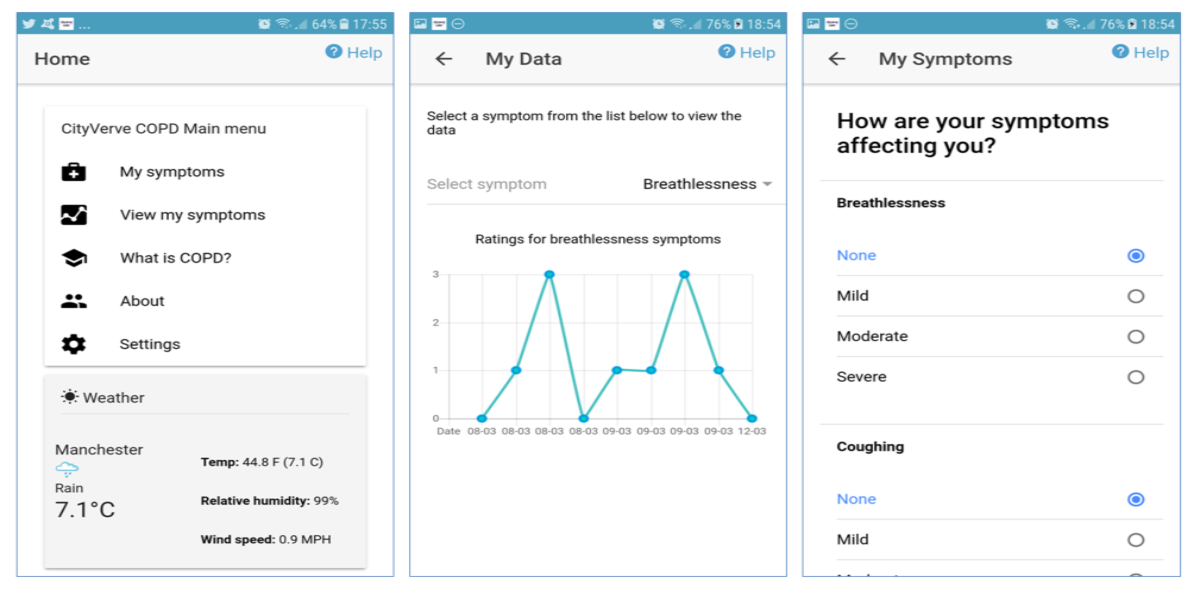
Screenshots of app: (left) home/landing page with the main menu and weather widget (center) graph showing ratings for breathlessness symptom and (right) self-reported impact of symptoms.

The app (Figure 1) was developed as a hybrid Web app using Apache Cordova and the Ionic framework and was designed to exploit the experience sampling method (ESM), which allows for the daily submission of data rather than retrospective completion, leading to potential memory bias [20]. Once downloaded, the app takes users through several setup screens. These screens collect some one-time data about the user (Textbox 2).

**Figure 2:**
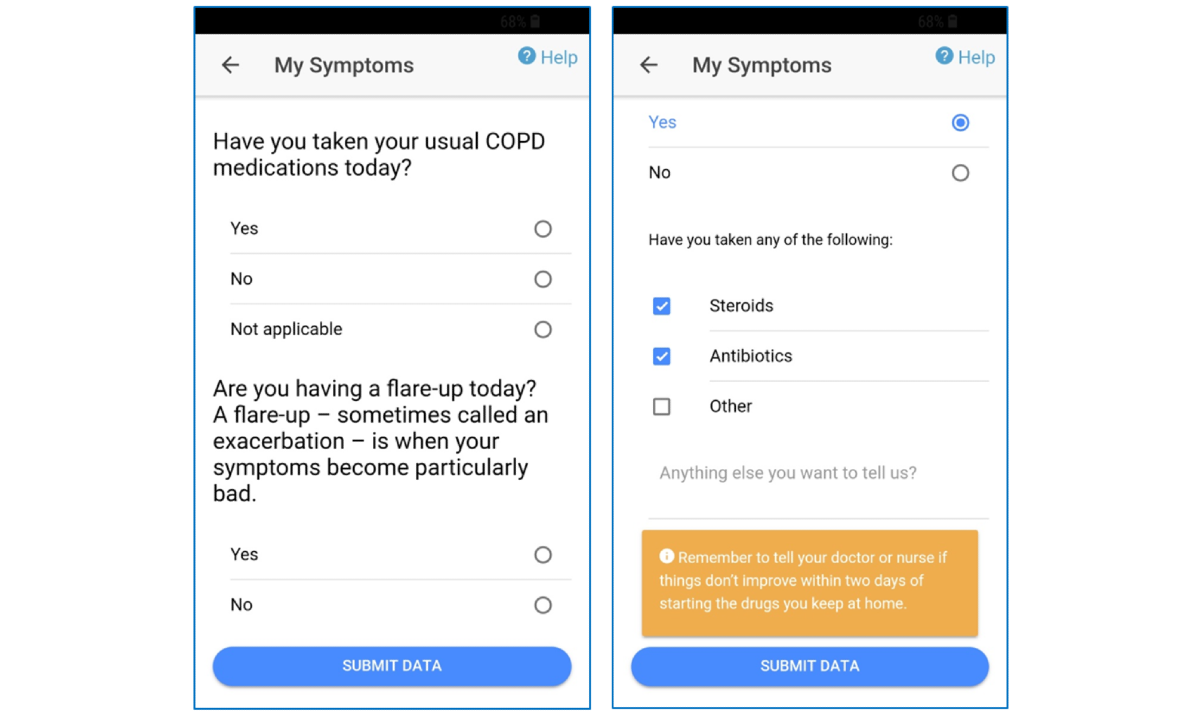
Screenshots of the app pages for medication questions and flare-up medications.

The main menu page displayed the current weather data, including temperature, humidity, and wind speed, as seen in the gray box in the first screen (home page) in Figure 1. The *My symptoms* page allowed users to record their general well-being, measured on a 3-point scale (*great*, *so-so,* and *bad*). Following this, they entered information about five key symptoms of COPD (breathlessness, coughing, mucus, tiredness, and sleep quality on a 4-point scale) and their medication use (Figure 2). The scales used to assess symptoms and well-being were adapted from the Britain Breathing app [21].

Participants could view their self-reported ratings for each symptom in the form of graphs presented on the *My data* page (Figure 1, center). This is accessed by tapping the *View my symptoms* menu option on the home page (Figure 1, left). Educational material about COPD was presented on the *what is COPD?* page. The *About* page contained information about the study and the participant information sheet. Each page had a help button displayed in the top right-hand corner, which included a video with an audio description of how to use that page.

**One-time setup data captured by the app (modifiable subsequently via the settings screen).**

- Gender (male or female)
- Year of birth
- Chronic obstructive pulmonary disease status (yes or no)
- Smoking status (yes daily, yes occasionally, no never, or no but I used to)
- Number of days they exercise for at least 30 min (0-14 or >14)
- Daily reminder for recording symptoms and medication (if relevant)

## Results

### Phase 1: User Engagement

**Figure 3:**
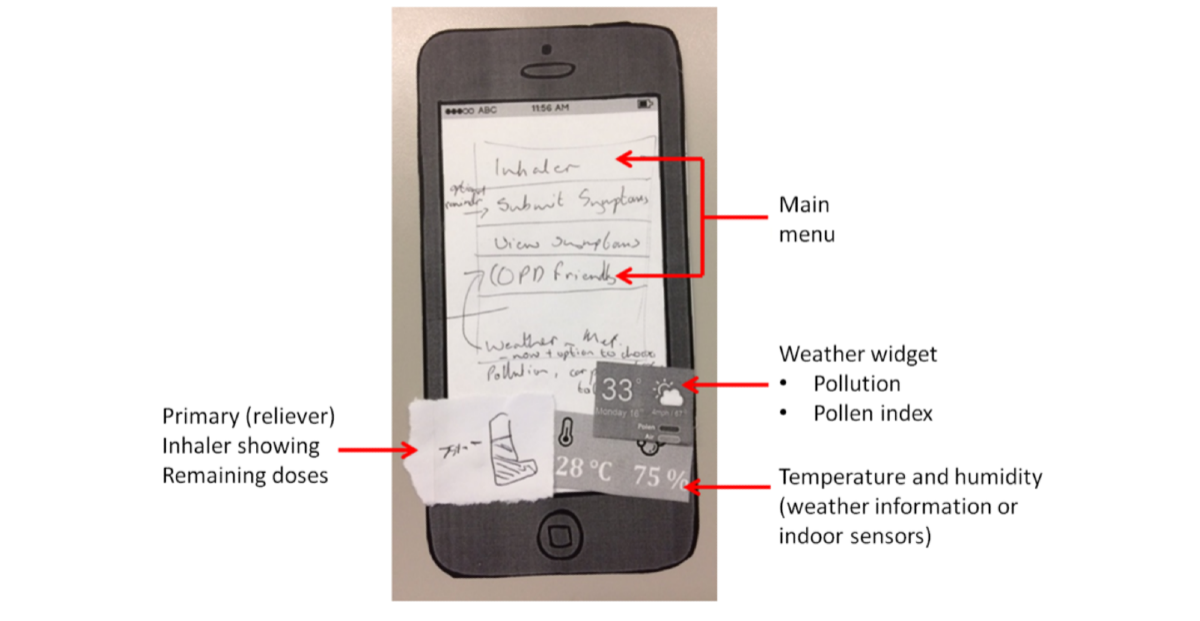
Homescreen mock-up.

Overall, users were enthusiastic about the concept of using an app to manage and monitor their condition. They welcomed the idea of being able to easily share information with family members, caregivers, and health care professionals, especially as several participants described difficulties communicating with doctors. Participants saw a value in the app for COPD patients in terms of recording, viewing, and monitoring symptoms, monitoring medication, and providing information about weather and characteristics of different locations to enable planning of activities. Figure 3 shows an example paper prototype developed with users.

#### Chronic Obstructive Pulmonary Disease–Friendly Map

Participants suggested the inclusion of a *COPD friendly map*, which could be utilized to display locations that were *friendly* to COPD patients. This would include being able to view COPD-relevant details, such as the presence of elevators, stairs, gradients, weather, air pollution, pollen, and air quality. They were interested in using this information to help them plan journeys to places that would cater to their individual health needs. It was not possible to implement this feature for the prototype app due to limited timescales. This was not deemed to be a core requirement of the app by the participants but rather a desirable but optional, additional feature. Given the technical demands of providing such a feature that would involve allowing users to mark features on maps and rate areas and sharing this information with each other, this was not implemented in the prototype app.

#### Smart Inhaler

Participants commented that a smart inhaler that links to the app would be useful for monitoring the remaining doses. They suggested that an inhaler icon should appear on the app home screen, which would indicate remaining levels of medication. Participants were also keen to obtain feedback on the effectiveness of their inhaler technique. Smart inhaler integration was not implemented in the prototype app owing to project time constraints.

#### Symptom and Medication Monitoring

Participants emphasized the utility of symptom monitoring due to the fluctuations in the condition leading to participants being unsure about how best to use their medication for symptom control. They described difficulties recalling these details during their infrequent medical reviews. They indicated a preference for a visual depiction of this information. On the basis of the patients’ recommendations, we selected five symptoms to be monitored within the app (breathlessness, coughing, mucus, tiredness, and sleep quality).

### Phase 2: Usability Study

**Figure 4:**
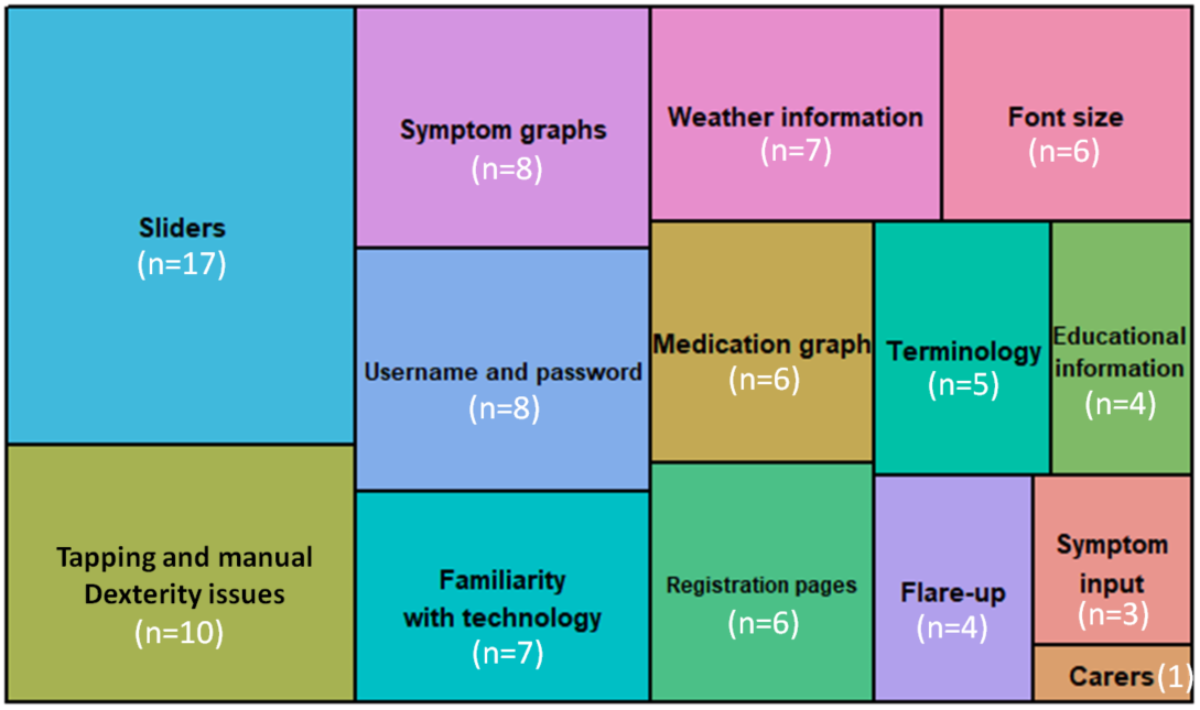
Treemap depicting the number of references made to each coded theme.

On the basis of phase 1, we developed a prototype that was tested with 5 people (4 females and 1 male) in the usability study. This included 4 people, who self-identified as having COPD, and 1 caregiver. The participants comprised 5 volunteers (4 females, all aged ≥55 years), and all of them had clinician-diagnosed COPD for more than 5 years. Participants were recruited through a respiratory patient support group and a general practice patient forum, and all of them had prior experience of being members of research advisory or stakeholder groups; hence, they were familiar with medical terminology. All but 1 participant used a smartphone at least occasionally. Moreover, 1 participant stated that she did not usually use a smartphone. Participants gave an average score of 32.4 out of 55 (SD 8.47) on the SUS, suggesting that they rated its usability as poor. The results of the qualitative analysis of the audio transcriptions and observational notes reveal the main issues faced by users. The main themes identified are shown in Figure 4. The number in brackets indicates the number of times that theme was mentioned.

#### App Information Displayed to Users

##### Weather Information

Participants were specifically interested in information about humidity, wind, and temperature changes. They also indicated that they would prefer weather information for a longer period to plan activities in the future:

it might be helpful if it could give you more than one day, the anticipated, so for instance you could think, oh, I don't think I better go shopping today I'll go tomorrow.

P3, COPD

This information was also considered useful for caregivers:

It's interesting for me to know that because if I look at it and see that it's anything like 90 or above I know he's not going to be in a fit state to do anything today, and I don't suggest going out or do anything, you know. So that's one of the places where a carer needs to know.

P4, caregiver

##### Educational Information About Chronic Obstructive Pulmonary Disease

When reflecting on experiences of their initial diagnosis, participants suggested that future COPD patients would want to know more about the nature of the condition and the prognosis. Participants particularly liked diagrams and felt that these were most helpful for understanding airway constrictions and how they cause symptoms:

I think the diagrams are very clear and good. They are helpful, and definitely show the changes that take place, and why, and it helps you think why you might get some of the symptoms that you do.”

P1, COPD

##### Information Feedback Level

A main feature of our experience sampling app involved feeding back information to users to enable them to track their symptoms and disease progression. We were interested in assessing which level of information would best suit participants. Participants were able to interpret the symptom graphs (Figure 1) displayed by the app accurately. This was elicited by directly questioning participants about their understanding of the graphs and what they represented:

Interviewer 2: “Okay, so can I just ask, on that graph, so what do you reckon it shows, when it goes up and when it goes down? What does that show?”

Participant 1: “When it goes up and down, like I’ve just said, the further it goes up, the more breathless I am, and for me, that’s how it would look for me, and when it’s down to zero, I’m not breathless at all.”

Interviewer 2: “Yeah, okay. And on there, on the horizontal axis?”

Participant 1: “The days, that’s fine.”

Participants preferred graphs to be simple, clean, and minimal. They made several suggestions to improve the appearance and interpretability of graphs, including avoiding the use of color alone, and ensuring graphs can be presented in a sufficiently large format on the screen.

One participant was unable to interpret the graph and expressed little interest in understanding symptom fluctuation with medication use:

Participant 5: “Well, that's your meds, isn't it, and that's your...Okay. [...] Yeah. But I don't know whether that would make any difference to me, I don't know whether it's relevant.”

Interviewer 2: “Mmm. What would you think? Looking at this graph, what would you think does it tell us about the medication in this case?”

Participant 5: “Well, the green one is medication not taken. No, that's the red one. Yeah. Obviously, the green one is taken. I don't know what it really means to me; it doesn't really mean anything.”

#### Terminology

Participants recommended using terminology commonly used by health care practitioners. They suggested that people with the condition would be familiar with *medical* terminology due to its frequent use by health care practitioners; they did, however, suggest including other commonly used synonyms for mucus, such as *phlegm* and *sputum*.

#### Usability Issues

Certain design features posed a challenge to participants due to manual dexterity issues, visual impairments, and unfamiliarity with smartphone usage. Participants frequently accidentally triggered functions within the app:

It didn't give me a chance to rate how I was feeling, just clicked straight over into how are your symptoms affecting you?

P1, COPD

Participants struggled, particularly, with typing due to the small size of the fields assigned to each letter within their smartphone's default virtual keyboard. This meant that security features such as inputting a unique username and password were challenging:

Aren't they small these? [tries to type username] Well that was supposed to say [NAME]. [...] But the size of your finger doesn't cater for these does it?

P3, COPD

These issues were exacerbated when coupled with visual impairments:

Yeah. Okay. Invalid password. My passwords don't match, that's because I'm not very good at hitting keys either. I have a stigmatism, which means I tend to go one to the right or one to the left, or that bit

P4, caregiver

Although most participants reported no issues with the font size, 1 participant reported that the font was *just about [her] limit* and suggested that being able to zoom in on the text would improve readability.

**Figure 5:**
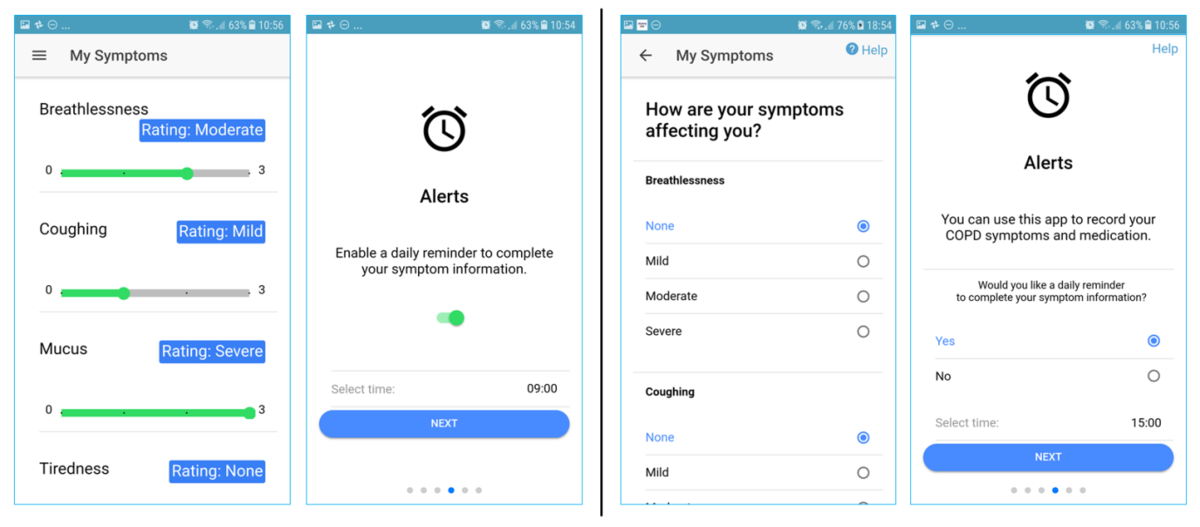
Screenshots of the app pages using slider controls (left of the vertical bar) before they were replaced following user feedback by a more explicit list structure (right of the vertical bar).

Manual and visual issues as well as lack of familiarity meant that certain design features common to many modern smartphone apps, such as sliders (Figure 5), were particularly challenging to participants:

It's hard for me because [...] I'm not used to sliding things, that's the bit I forget to do.

P4, caregiver

The toggle switch (Figure 5) proved particularly challenging for users. Users were unsure how to engage with this function (tap or slide), struggled with the small size, and struggled to interpret its meaning (the slider turned grey when switched off and green when switched on, as is common in many smartphone apps). Participants preferred large buttons that they could *hit properly*:

Interviewer 2: “So, can I just ask, does that mean you prefer not to have a daily reminder?”

Participant 1: “No.”

Interviewer 2: “Or would you like to have one?”

Participant 1: “Yes, the daily reminder would be good*.*”

Interviewer 2: “Okay.”

Participant 1: “So, the green means that it's set to remind?”

Participants also appeared to prefer clearly labeled buttons, rather than implicit symbols or color coding:

Participant 5: “Yeah. Enable a daily reminder to complete your symptoms and information. We did that, but it was only when you asked me if I didn't want a reminder. [...] The fact that I wouldn't have done if I hadn't have asked you what you wanted.”

Interviewer 1: “Right.”

Interviewer 2: “So would it maybe be more useful if it said something like, do you want a daily reminder, and then there would be yes or no, and you'd click yes or no?”

Participant 5: “Yeah, exactly.”

#### Technology Support and Help Requirements

Our observations revealed that participants may require help and support with basic aspects of smartphone usage, such as instructions on how to tap on items using the fingertip, as the following interaction highlights:

Participant 3: [tries selecting an item on screen using fingernail]

Interviewer 1: “Can I suggest that if you use your finger rather than your nail because that's...”

Participant 3: “It's also put the wrong date of birth in. It doesn't like me.” [tries to select item on screen by pressing the fingertip down and holding]

Interviewer 2: “I think if you try...”

Interviewer 1: “Shorter. Yeah, just a bit shorter, yeah, there we go.”

Several users appeared to search for help functions when they were unsure how to proceed, and they expressed a need for additional support and instruction:

They don't tell you where any of these things, it's not just you, they don't tell you where to tap. Do you have to tap on female or do you want it tapped on the dot at the end? That might make a difference.

P3, COPD

#### Familiarity With Mobile Technology

All participants reported low familiarity and confidence in smartphone usage:

You're dealing with a complete technophobe here.

P1, COPD

I presume that's a phone is it?

P3, COPD

#### Validity of Participant Feedback

In assessing participants’ feedback regarding the app against the researchers’ observation notes, some discrepancies became evident. For example, observation notes indicate that participant 1 struggled with the slider controls, tending to tap rather than drag the control, and switching functions off when they intended to switch them on. When asked their opinion regarding the sliders, the participant nevertheless replied:

It's nice, it's not bad to use.

Following feedback from the usability study, design changes were made to mediate the issues identified. These included the following:

- Adding a help feature (help videos)
- Replacing the sliders with clearly labeled option lists (Figure 5)
- Improving the size and readability of text and input fields.

**Figure 6:**
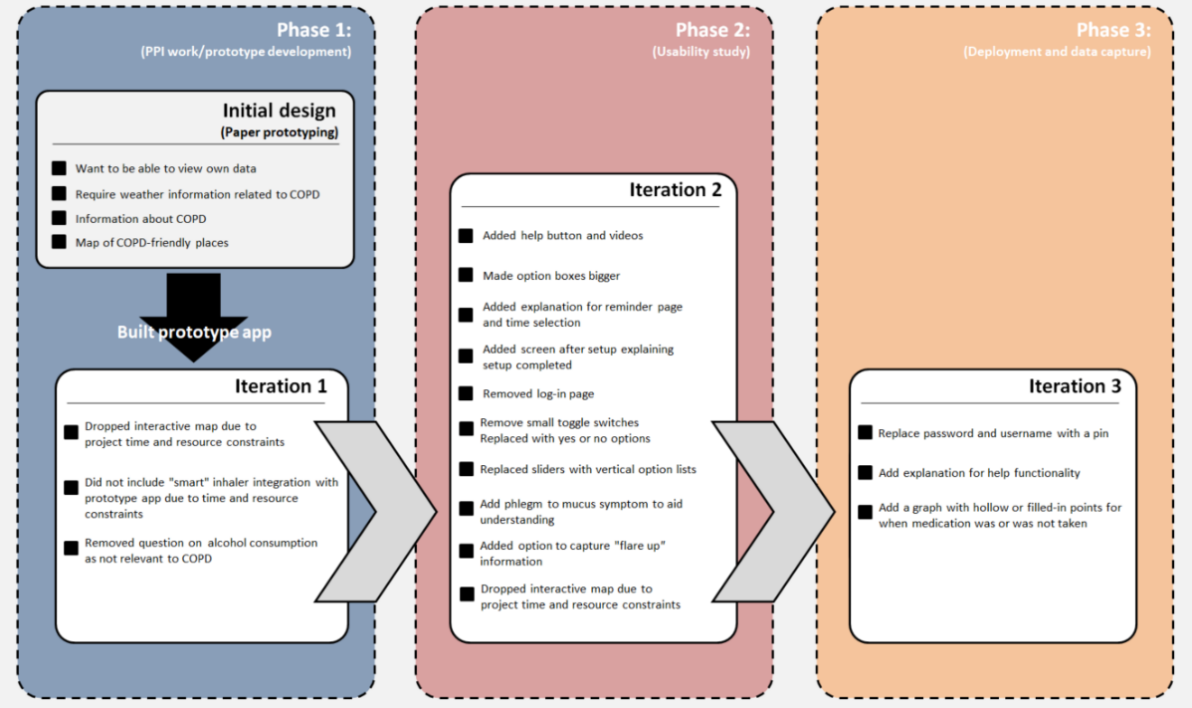
Iterative design and development phases. COPD: chronic obstructive pulmonary disease.

Figure 6 highlights the entire iterative design and development phases, including the key changes made in each iteration of the app leading to the final changes in iteration 3 suggested for the final *deployment* version of the app.

### Phase 3: Pilot Testing Phase

#### Experience Sampling

In addition to the two devices the app was directly installed on during the UE, a further 41 users downloaded the app from the Google Play store. Moreover, 48% of the downloads came from the United Kingdom and 36.59% from the United States. A total of 37 participants used the app between May 2018 and August 2018; median age of the participants was 47 years (mean 45.2 years, SD 23.8 years). Table 1 shows participants’ demographics. In 83% (31/37) of cases, participants identified as having a diagnosis of COPD and 16% (6/37) identified as not having a COPD diagnosis. A total of 25 users entered the data only once.

**Figure 7:**
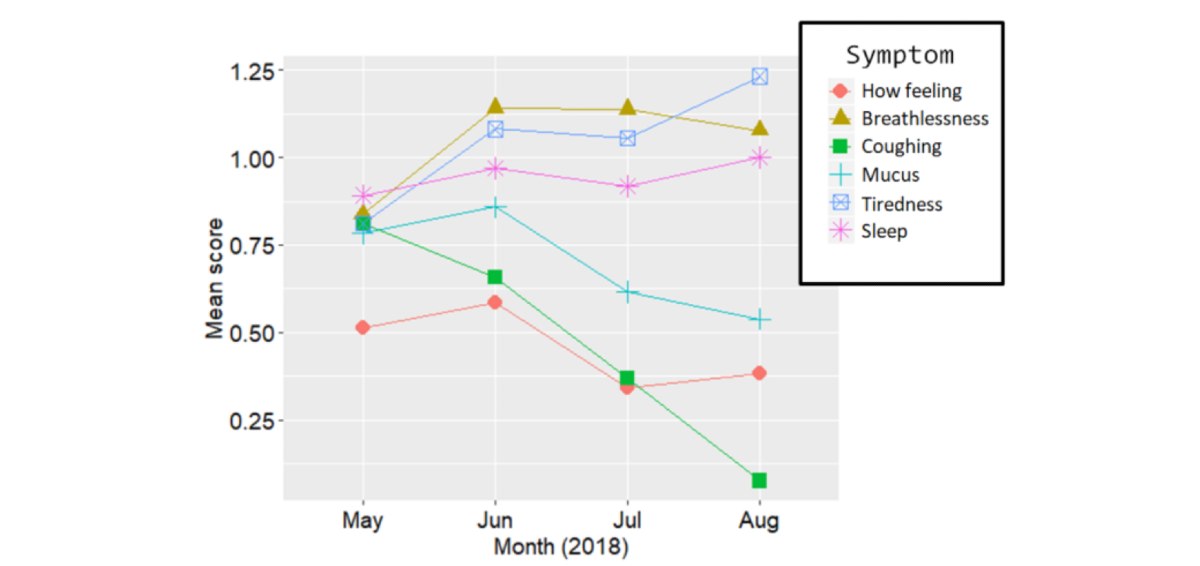
Average score for all participants (N=37) per month for the impact of symptoms. Note that "how feeling" is rated on a 3-point scale (0=great, 1=so-so, and 3=bad), and the other symptoms are rated on a 4-point scale (0=none, 1=mild, 2=moderate, and 3=severe).

Figure 7 shows participants’ symptom ratings over the 4-month period. The symptoms most correlated to perceived wellness coded with the variable *howFeeling* were *tiredness* (*r*=0.61; *P*<.001; 95% CI 0.51-0.68) and *breathlessness* (*r*=0.59; *P*<.001; 95% CI 0.49-0.66). The average symptom ratings over the 4-month period (Table 2) show that the reported impact was highest for tiredness and breathlessness. Due to the small sample size and variation in symptom reporting (discussed in the Event Data section) in the pilot data, we would need to collect more data points for a longer period to carry out a more well-informed subsequent analysis. This should also account for seasonal variations in symptom reporting. Currently, these data are insufficient to draw inferences from, as the increased spread of symptoms over time may be related to the decrease in data points over time or other unknown factors. The subsequent analysis of the number of users reporting symptoms and the frequency of symptom reporting was important as it helped to provide some context to the data presented in Figure 7 (eg, being able to see that the majority of consistent reporting data were derived from only 3 participants, despite 37 people having recorded their symptoms at least once).

**Table 1:** Demographic data self-reported by participants (N=37).

Demographic		Value, n (%)	
**Gender**			
	Male		23 (62)
	Female		14 (37)
**Self-identified COPD^a^** **status**			
	Has COPD diagnosis		31 (83)
	Has no COPD diagnosis		6 (16)
**Smoking status**			
	Yes, daily		4 (10)
	Yes, occasionally		8 (21)
	No, never		4 (10)
	No, but I used to		21 (56)
**Exercise frequency**			
	1 day per week		10 (27)
	2 days per week		9 (24)
	3 days per week		9 (24)
	4 days per week		4 (10)
	5 days per week		3 (8)
	6 days per week		2 (5)

^a^COPD: chronic obstructive pulmonary disease.

**Table 2:** Average rating for each symptom for all participants (N=37) over a 4-month period.

Symptom	Value, mean (SD)
Perceived wellness (How feeling)	0.481 (0.59)
Breathlessness	1.085 (0.76)
Coughing	0.554 (0.79)
Mucus	0.747 (0.78)
Tiredness	1.036 (0.99)
Sleep quality	0.941 (0.75)

#### Event Data

To gain a deeper understanding of how users were using the app outside of a controlled setting, we deployed an event capturing system into the app [22]. Event data were then extracted and analyzed using a combination of the R [23] package *bupaR v0.4.0* and the *WevQuery* tool [24] to apply pattern mining to the event data.

**Figure 8:**
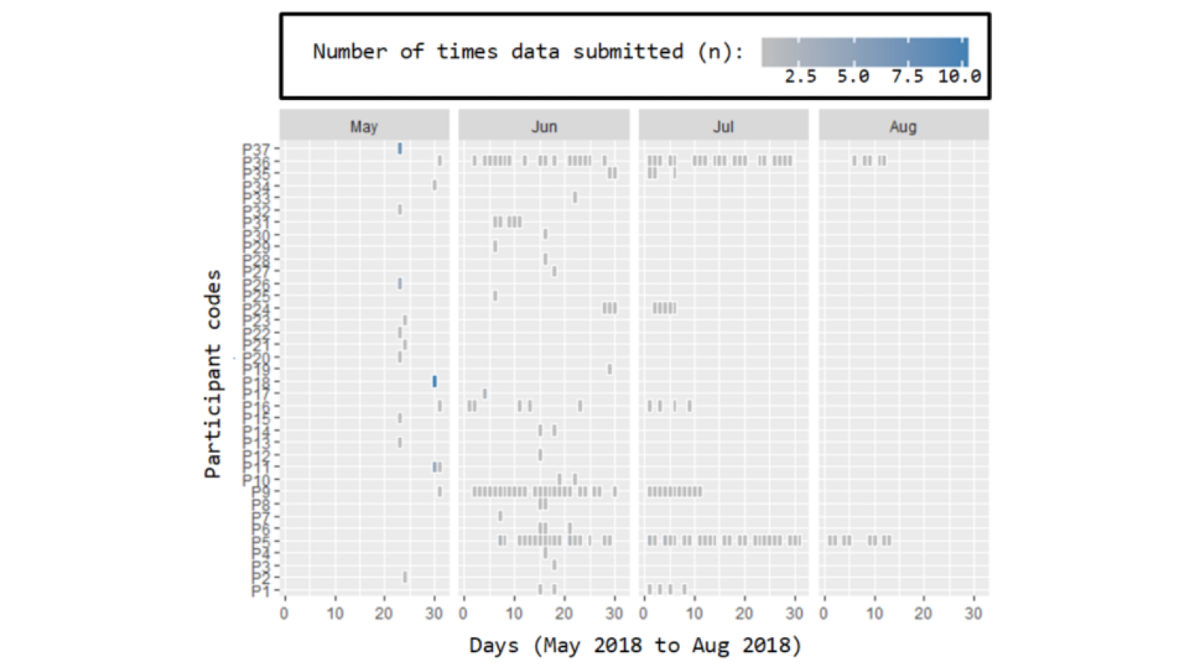
The number of times each participant submitted his or her self-reported symptom data per day per month.

Figure 8 shows the number of times data were submitted by each participant over the 4-month period. This suggests a high dropout and low usage curve, with the exception of 3 participants who entered their symptom data on a regular basis (P36, P9, and P5). A total of 21 participants entered their data only once. Moreover, 2 participants (P18 and P37) exhibited unexpected behavior and entered their data 10 and 7 times in a single day, respectively, and did not enter any subsequent data.

**Figure 9:**
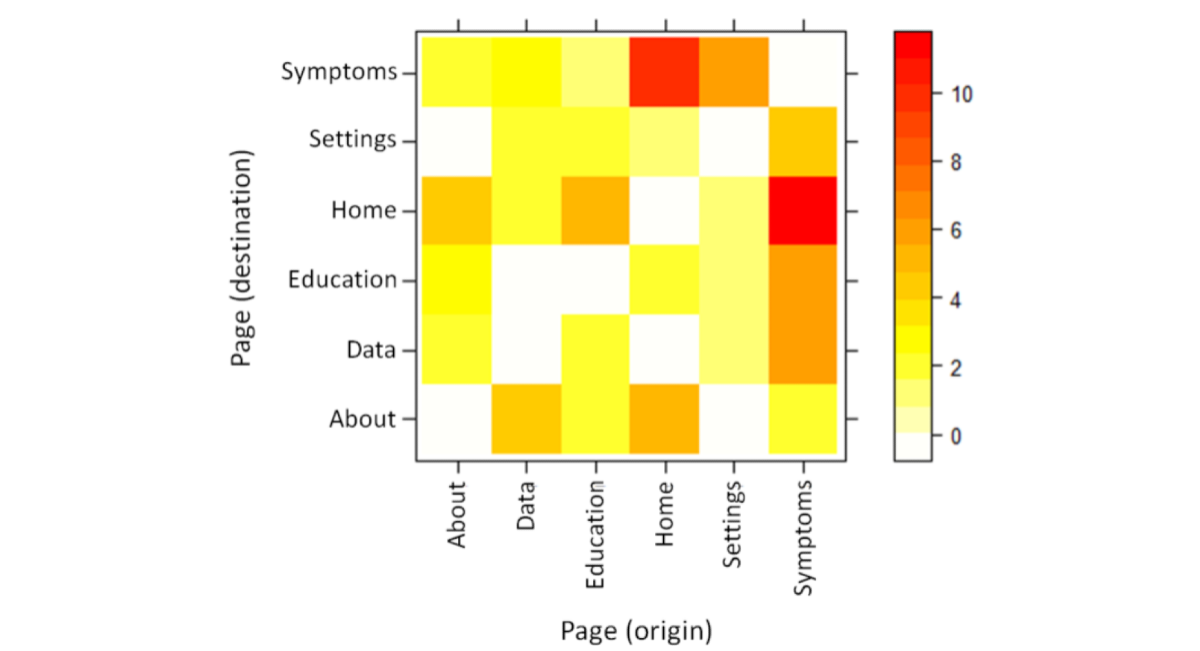
Most common sequence of events within an episode between pages.

The help button that appears on each page was selected by 4 users a total of 8 times. Figure 9 and Textbox 3 highlight the most common sequences of events between pages. This shows that, as intended, the symptom logging and viewing of data are among the most common sequences, suggesting that users viewed their data regularly after submitting it.

**Common mouse down event sequence patterns.**

Mouse down event patterns

- Menu (symptoms page button) —> symptom page —>medication page (n=173)
- Menu (view my data button; n=86)
- Symptom page —> menu (view my data button; n=51)
- Medication page —> menu (view my data button; n=51)

## Discussion

### Principal Findings

We found that many of the issues affecting our work with participants were similar to those faced by O'Connor et al [25], who attempted to co-design an app with dementia patients and their caregivers, including unfamiliarity with technology and incorrect perceptions about how users would interact with the technology. This led to the initial design ideas being significantly revised. So far, very little qualitative research regarding the perception of patients and their caregivers and how they experience technology has been carried out [26]. The findings of our study reinforce the importance of considering the unique users of the technology we are proposing. When working with people with chronic diseases, their age, education, and Information Technology literacy should be taken into account [2]. This is especially relevant for the COPD population.

Many design features commonly used in mobile apps are hitherto unknown to the COPD population, such as the sliders that were initially included in the app. Our research indicates that such design elements are not optimal for a COPD population. Previous research corroborates this by showing that older users require larger touch targets (minimum 15×15 mm) with sufficiently large gaps between touch targets (minimum 6 mm), ideally complemented through support functions such as speech input [27].

Besides the physical impairments, our study highlights the importance of considering lack of familiarity and confidence in technology use. Our observations as well as participants’ verbalizations while navigating the app suggest that older users may require more help functions within apps that provide support regarding app usage as well as general smartphone usage (eg, how to tap items). Research shows that among those older than 65 years, the majority do not feel confident in using computers, smartphones, and other electronic devices [28].

We used ESM to capture patients’ symptom information, as it has been shown to provide an adaptive and personalized system for the monitoring and adaptation of treatment strategies as well as increasing ecological validity and reducing memory biases [20,29]. ESM has also proven to be a useful method in other conditions where the impact of symptoms varies over time [21]. Information about COPD was also included in the app. This was introduced following input from patients attending lung events, before app construction, as several patients highlighted a lack of information from their general practitioner about the condition.

Our study highlights that physical impairments and lack of familiarity can particularly affect initial registration processes, such as creating usernames and passwords. This step is crucial for health-related apps, as password protection is needed to protect sensitive data. On the basis of our findings, we recommend that this process be kept brief and simple, for example, by using a simple 4-digit pin. Registration pages should also be supported through a feedback system to identify errors immediately and reduce user frustration [27].

Given the demographic qualities of the COPD population, the development of apps for this target population may be reticent to present information in the form of graphs; however, our research suggests that graphs can be interpreted correctly and perceived as useful if they are presented in a simple, clear format. Previous research confirms that graphics and multimedia should be used sparingly and purposefully when targeting older users, and text alternatives should be provided where possible [27].

Another important insight from our work reflects on the validity of user feedback. Features that users appeared to struggle with according to observation notes were nevertheless described as *fine* by users, suggesting a social desirability bias [30]. This underscores the importance of developing apps not only based on users’ verbalizations and responses to specific questions (such as standardized usability scales) but on observations of user behavior while engaging with the app. This limitation has been noted in previous usability evaluations [31,32] and requires due consideration during app development.

Attrition rates are a known issue in digital health interventions [33]. The high level of attrition seen in our study is, therefore, not entirely unexpected. An understanding of why people discontinue use is important and worthy of further research given that retention is key for the management of chronic conditions over time [33]. This is especially challenging as chronically ill people may experience *diary fatigue* and be unlikely to keep accurate records of their condition, especially when unwell [34,35]. Another factor that may have contributed to the lack of use is that the reminder notification did not work on all devices due to an issue with the local notifications plugin path in a package of the Ionic framework.

The usage data indicate that the app was used as intended with most of the activity surrounding the recording and viewing of symptom data, which was the app’s principal purpose. The low usage of the *help* feature may suggest that the app was also fairly intuitive for most people. The biggest issue surrounds the lack of continued use of the app over time. More research is needed to identify the intrinsic and extrinsic reasons behind the attrition in this context. Longer-term analysis of app usage before dropout using *in the wild* (deployed in the real world) event capture may help to shed light on some of these reasons.

### Design Implications

As a result of the usability work carried out with participants, we can infer several considerations for designing COPD support apps, including the following:

- Large font size
- Large clearly labeled input (eg, buttons)
- Avoid items requiring greater tactile manipulation than tapping (ie, sliders)
- Provide easy to access help feature on each screen
- Label items clearly; do not rely on intuitive features
- Include the ability to zoom into content
- Passwords can be difficult; consider reducing the required length and inclusion of special characters.

### Chronic Obstructive Pulmonary Disease–Specific Implications

The implications specific to those with COPD include:

- Information about weather was especially welcomed by participants, given its effect on the condition. Participants were keen to have everything in one place (app) rather than using multiple different sources of information.
- Future apps should consider implementing
  COPD-friendly maps
  and linking to smart inhalers to show remaining medication levels and feedback regarding usage techniques.
- Participants valued having information about the condition in the app, as many highlighted during PPI events that they often received little or no information following diagnosis. They valued easily accessible (not too technical) and reliable information.
- When developing disease support apps, discussing terminology with patients and their health care providers to determine which terminology they usually use is advisable. It may be acceptable to use medical terminology, as long as this is commonly used. In some cases, this may be more beneficial than trying to use lay terms. However, this may be restricted to those with a longer history of COPD who have had regular interactions with health care professionals regarding their COPD.

### Recommendations

**Figure 10:**
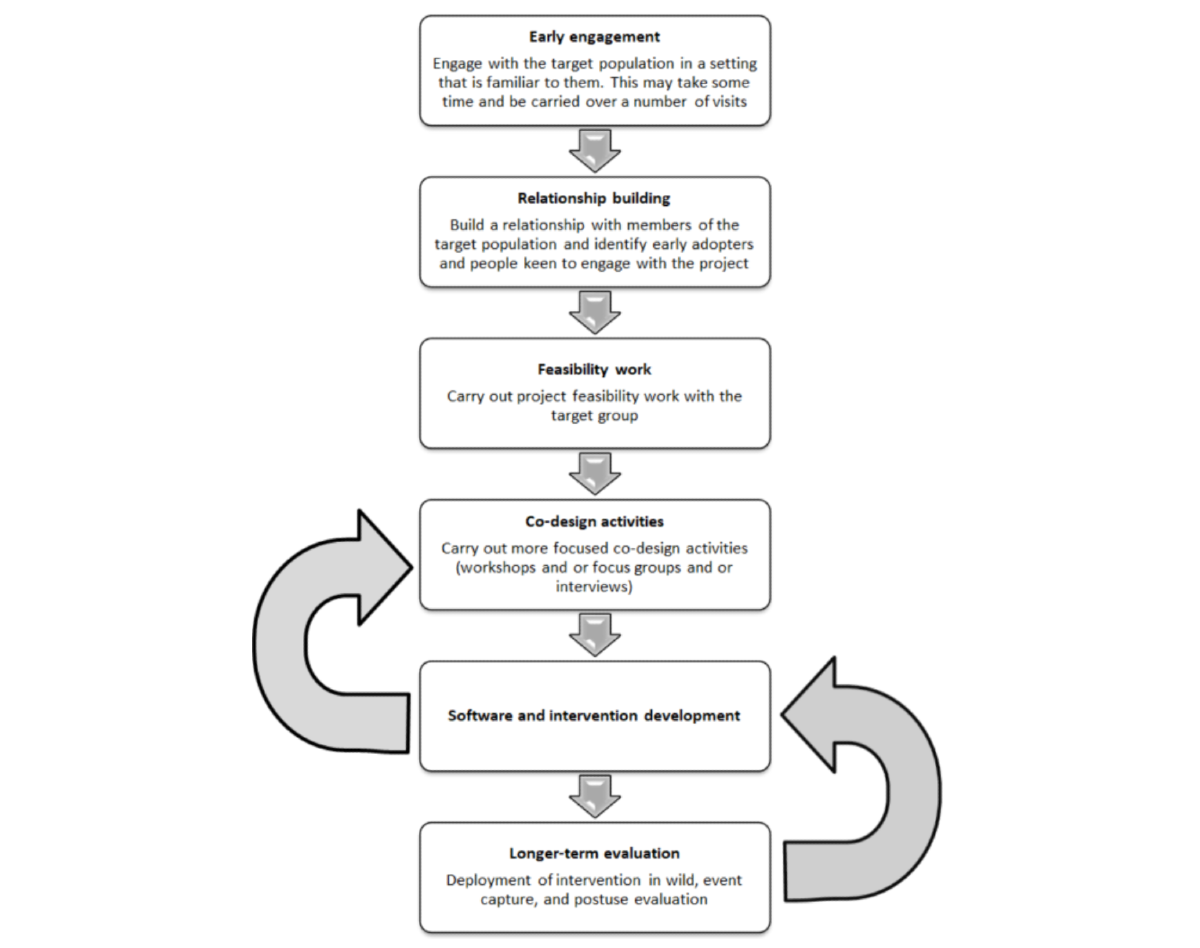
Recommended steps.

The principal recommendation based on our experience of working with a challenging target population on a large project with multiple stakeholders is that the population of interest, researchers, and developers should all be involved at every stage of the project and that an iterative approach be used to build a prototype. This prototype should then be tested in a wider environment with a larger group of the target population, where their interaction with the app is evaluated over a longer period to determine further issues and acceptability of the final intervention. The main stages are summarized in Figure 10.

### Limitations

Our PPI work involved participants from COPD support groups who expressed an interest in building a smartphone app. Such participants are more likely to be educated about COPD and may have more familiarity with technology than the standard COPD user. We attempted to mitigate this by capturing patterns of event behavior (ie button presses and transitions) from a wider group of users post deployment. There is an argument that such interventions in general do not adequately address the *digital divide*, as only those with access to such interventions in the first place may benefit. This rules out many older and low-income users [36]. Moreover, as our usability study was conducted with individuals who had a COPD diagnoses of more than 5 years’ duration, findings may not adequately represent those who were recently diagnosed. In addition, the time of year is likely to have had an impact on the reported symptom frequency and severity. Finally, it should be noted that the 3-point scale for well-being used in our app is not a validated measure. It was developed based on user engagement and has high face validity, but it is not clear how well it correlates with actual well-being.

### Conclusions

We found that working with members of the target population at all stages of the project was a useful strategy; stakeholder engagement aids the development of research interventions that are both adaptive to the needs of the patient and the preferences of the provider [37]. This is different from a traditional researcher-led approach or a pure software engineering approach, such as *agile*. Placing the target population at the center of the work and iteratively building the intervention with the target users allows for the creation of a more acceptable final product. The combination of qualitative data analysis and data collected from open-source event capture tools also served to offer a further insight into app usage and dropout. We must try where possible to mitigate bias when carrying out such work, including social desirability bias and biases associated with participants who display more familiarity with technology than the typical target end user.

## Appendix

Multimedia Appendix 1 Example of framework analysis.

## Abbreviations

COPD: chronic obstructive pulmonary disease
ESM: experience sampling method
mHealth: mobile health
PPI: patient and public involvement
RCTs: randomized controlled trials
SUS: System Usability Scale
