# Supplementary figures and images for: Recommendations for Developing Support Tools With People Suffering From Chronic Obstructive Pulmonary Disease: Co-Design and Pilot Testing of a Mobile Health Prototype
Source: JMIR Hum Factors. 2020 May 15;7(2):e16289. doi: 10.2196/16289 (PMC7260664; doi:10.2196/16289)

# Appendix

Example of framework analysis.


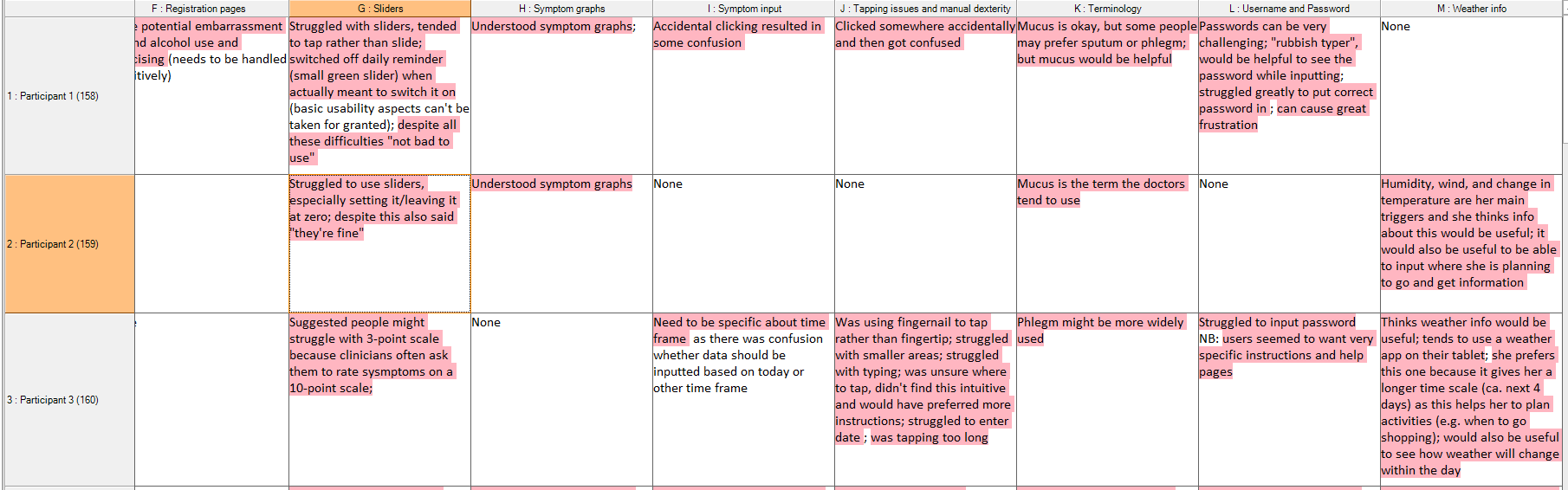

Supplement: Multimedia Appendix 1 [file humanfactors_v7i2e16289_app1.docx]
